# The Role of Histopathology as a Complementary Diagnostic Tool in the Monitoring of Bovine Tuberculosis

**DOI:** 10.3389/fvets.2022.816190

**Published:** 2022-05-13

**Authors:** Fernanda Larenas-Muñoz, José M. Sánchez-Carvajal, Ángela Galán-Relaño, Inés Ruedas-Torres, Eduardo Vera-Salmoral, Lidia Gómez-Gascón, Alfonso Maldonado, Librado Carrasco, Carmen Tarradas, Inmaculada Luque, Irene M. Rodríguez-Gómez, Jaime Gómez-Laguna

**Affiliations:** ^1^Department of Anatomy and Comparative Pathology and Toxicology, University of Córdoba, International Excellence Agrifood Campus ‘CeiA3’, Córdoba, Spain; ^2^Department of Animal Health, University of Córdoba, International Excellence Agrifood Campus ‘CeiA3’, Córdoba, Spain

**Keywords:** bovine tuberculosis, histopathology, diagnostic tests, tuberculosis like lesions, sensitivity, specificity

## Abstract

The diagnosis of bovine tuberculosis (bTB) is based on the single intradermal tuberculin test (SIT), interferon gamma, and compulsory slaughter of reactor animals. Culture and PCR from fresh tissue are regarded as gold standard techniques for *post-mortem* confirmation, with the former being time-consuming and presenting moderate to low sensitivity and the latter presenting promising results. Histopathology has the advantage to identify and categorize lesions in both reactor and non-reactor animals. Therefore, this study aims to highlight the role of histopathology in the systematic diagnosis of bTB to shorten the time to disclose positive animals. Blood (212) and lymph node (681) samples were collected for serological, bacteriological, and histopathological analyses from a total of 230 cattle subjected to the Spanish bTB eradication program. Seventy-one lymph nodes and 59 cattle yielded a positive result to bacteriology, with 59 lymph nodes and 48 cattle presenting a positive result in real-time PCR from fresh tissue. Roughly 19% (40/212) of sera samples gave a positive result to ELISA. Tuberculosis-like lesions (TBLs) were observed in 11.9% (81/681) of the lymph nodes and 30.9% (71/230) of cattle. Noteworthy, TBLs were evidenced in 18 out of 83 SIT^−^ and real-time PCR and bacteriology negative animals, with 11/18 disclosing a positive result to Ziehl-Neelsen technique and two of them to ddPCR from paraffin blocks targeting IS*6110*. Six out of these 11 ZN^+^ corresponded with mesenteric LN and were confirmed positive to paratuberculosis. Histopathology yielded a sensitivity of 91.3% (CI_95_ 83.2–99.4%) and a specificity of 84.4% (CI_95_ 78.6–89.3%) with good agreement (κ = 0.626) when compared with real-time PCR. Our results confirm that histopathology allows a rapid confirmation of real-time PCR and bacteriology, emphasizing its contribution to bTB control and monitoring.

## Introduction

Bovine tuberculosis (bTB) is a chronic infectious zoonotic disease mainly caused by either *Mycobacterium bovis* or *M. caprae*, and other members of the *Mycobacterium tuberculosis* complex (MTC) that affects various species of mammals (1). BTB is a significant economic threat to farm businesses in the form of trading restriction and slaughtering of reactor animals within the eradication campaign framework (1, 2). In many countries, the eradication program has faced important challenges in its struggle against bTB due to the persistence of non-reactive positive animals in the farm and wildlife reservoirs, limiting their progress toward the eradication of this disease from cattle, the main source of infection for humans (1, 3). Therefore, the development of rapid and accurate diagnostic techniques and protocols for the detection and culling of MTC-infected animals is one of the cornerstones of success to control and eradicate bTB in cattle and other species (4–6).

In the European Union (UE), the current bTB control program is mostly based on the single intradermal tuberculin test (SIT) and interferon gamma release assays to disclose reactor animals, followed by compulsory slaughter of these animals and livestock movement restrictions measures (7). The microbiological culture has been traditionally considered as the gold standard technique for bTB diagnosis (8, 9). However, it is reported as a time-consuming technique with a moderate to low sensitivity (Se), delaying the turnaround time to make a decision for 3 months (9).

Nowadays, the diagnostic tests available for bTB are imperfect because of limited Se and specificity (Sp) (5, 10, 11). The standard interpretation of SIT leads to a Se of 68–96.8% and a Sp of 75.5–98.8% (11), while the gamma interferon test presents a higher estimated Se, between 73.0–100%, and Sp ranging from 85.0 to 99.6% (11, 12). BTB-ELISA test, validated by World Organization for Animal Health (OIE), presents a high variability of Se, ranging from 45.0 to 98.6% and Sp from 52.5 to 100% (13), although better estimates have been reported with alternative cocktails of antigens or different methodological approaches (14). In the case of *post-mortem* techniques, bacteriology presents average Se and Sp values of 78.1% (72.9–82.8%) and 99.1% (97.1–100%), respectively (15). On the other hand, real-time PCR directly from fresh tissue samples has been recently reported as a promising diagnostic tool with a moderate to high values of Se and Sp (16, 17).

Histopathology has the advantage to identify and categorize lesions in both reactor and non-reactor animals even when they are unnoticed during the visual inspection at the slaughterhouse. This technique presents an average Se of 93.6% (89.9–96.9%) for the diagnosis of bTB but with less Sp than other techniques, on average 83.3% (78.7–87.6%) (16). This approach must be followed by a definitive diagnosis that is dependent on the isolation of *M. bovis*, but it is worth to note that the use in parallel of histopathology and culture of lesion has shown to provide an effective way of identifying infected animals (15). Moreover, identification of microscopic lesions in no grossly affected tissues or tuberculosis-like lesions (TBL) in anergic animals can be achieved by means of histopathology. In this sense, a rapid confirmation of bTB in tuberculosis-free herds is pivotal to prevent the spread of the disease, with the combination of different diagnostic tools representing a smart approach to obtain an efficient diagnosis improving its control. Therefore, this study aimed (i) to highlight the role of histopathology in the systematic diagnosis of bTB to shorten the time to disclose positive animals, but also (ii) to evaluate the histopathology as a complementary tool to be used with real-time PCR targeting IS*6110* and microbiological culture to confirm positive animals.

## Materials and Methods

### Study Population

A total of 230 cattle, subjected to the surveillance and monitoring for bTB in the framework of the Spanish national eradication program (18) between October 2018 and December 2019 were included in this study. According to their SIT test result, the 230 cattle were classified as SIT positive (SIT^+^, bovine PPD ≥ 4 mm or SIT inconclusive 2 mm > bovine PPD > 4 mm) and SIT negative (SIT^−^, ≤ 2 mm bovine PPD), belonging to each group of 139 (60.4%) and 91 (39.6%) animals, respectively. SIT inconclusive results were considered as positive.

Blood samples and samples from retropharyngeal, tracheobronchial, mediastinal, and mesenteric lymph nodes (LNs) were collected along the slaughter line and kept refrigerated until arrival to the laboratory. Tracheobronchial and mediastinal LNs were processed as a pool (tracheobronchial-mediastinal LN) since volume sample was not enough when independently processed. Due to the logistics of the slaughterhouse and the timing of slaughtering, it was not always possible to collect blood and LN samples from the 230 cattle. Thus, a total of 212 blood samples for the serological analysis and 227, 226, and 228 samples from retropharyngeal, tracheobronchial-mediastinal, and mesenteric LNs, respectively, were collected. From each LN, samples were divided in two parts. One part was fixed in 10% neutral-buffered formalin for the histopathological analysis and the second part was processed to obtain a tissue homogenate for bacteriological culture.

### Bacteriological Culture

Every LN sample was individually homogenized with a tissue homogenizer (Fisherbrand, Fisher Scientific, Madrid, Spain) to obtain a uniform mixture and stored at −20°C until use (17). Briefly, 4–7 g of each LN tissue sample were placed into a 15 ml tube (Falcon^TM^; Corning, Madrid, Spain) with the same volume (w/v: 1/1) of 0.85% sterile NaCl and grinded until a homogeneous mixture was obtained. Tissue homogenate was used for selective bacterial culture according to the protocol carried out in a previous study (17). Briefly, the homogenate was decontaminated with an equal volume of 0.75% (w/v: 1/1) hexadecyl pyridinium chloride solution in agitation for 30 min. Samples were centrifuged for 30 min at 1,500 × *g*. Pellets were collected with swabs and cultured in liquid media (MGIT^TM^ 960; Becton Dickinson, Madrid, Spain) using an automatized BD Bactec^TM^ MGIT^TM^ System (Becton Dickinson, Spain). Culture was considered positive when isolates were identified as MTC by real-time PCR (19). Individually, an animal was considered positive when at least one out of the three LN samples was positive.

### Real-Time PCR From Fresh Tissue Targeting IS*6110*

Deoxyribonucleic acid extraction from homogenized tissue samples and subsequent real-time PCR targeting IS*6110* was performed in a parallel study from our research group (17). Briefly, DNA Extract Vacunek (VK; Bizkaia, Spain) was performed according to manufacturer's instructions with modifications. Specific primers (IS*6110*-forward: 5′-GGTAGCAGACCTCACCTATGTGT-3′; IS*6110*-reverse: 5′-AGGCGTCGGTGACAAAGG-3′) and a probe (IS*6110*-probe: 5′-FAM-CACGTAGGCGAACCC-MGBNFQ-3′) targeting a conserved region of IS*6110* transposon were used for the real-time PCR (16, 17, 20) by using the QuantiFast® Pathogen PCR + IC Kit (QIAGEN, Hilden, Germany). Real-time PCR was carried out using the QuantiFast® Pathogen PCR + IC Kit (QIAGEN, Hilden, Germany) with amplifications run in duplicate in the MyiQTM2 Two-Color qPCR Detection System (Bio-Rad, Hercules, CA, USA) under the following cycling conditions: 95°C for 5 min, followed by 45 cycles of 95°C for 15 s and 60°C for 30 s. Following the manufacturer's guidelines, an exogenous inhibition heterologous control (internal amplification control, IAC) supplied with the kit was included. Complete inhibition of amplification was considered when IAC did not amplify, while partial inhibition was considered when it showed a quantification cycle (Cq) > 33. An inter-run calibrator with a known Cq value of 32 was introduced in each assay to self-control intra-assay repeatably and accuracy. The limit of detection for this real-time PCR is from 10 to 100 genomic equivalents, and the cut-off is established at Cq <38 (17).

### Blood Samples and Antibody Detection

Sera was obtained from blood by centrifugation at 6,000 × *g* for 10 min and stored at −20°C until analysis. The presence of specific antibodies against *M. bovis* was determined by using a commercial ELISA kit (IDEXX, *Mycobacterium bovis* Antibody Test kit, Westbrook, Maine, USA) following manufacturer's instructions.

### Histopathology

Formalin-fixed LNs were routinely processed and embedded in paraffin. All samples were stained with hematoxylin-eosin (H&E) for their histopathological analysis. Histopathological findings were microscopically evaluated, and samples were classified as positive if TBLs were observed. TBLs were considered as those consistent with tuberculous granuloma, pyogranuloma, or scattered Langhans-type multinucleated giant cells (MNGCs). Tuberculosis (TB) granuloma were classified into 4 different stages as previously reported (21). Briefly, stage I (initial) granulomas are characterized by the presence of abundant epithelioid macrophages with lymphocytes, neutrophils, and, at times, MNGCs. Stage II (solid) granulomas are characterized by epithelioid macrophages surrounded by a thin connective tissue capsule. Infiltrates of neutrophils and lymphocytes may be present along with MNGCs. In addition, the necrosis, if present, is minimal. Stage III (necrotic) granulomas are characterized by a necrotic center surrounded by a zone of epithelioid macrophages with or without MNGCs and lymphocytes and encapsulated with fibrous connective tissue. Stage IV (necrotic and mineralized) granulomas are characterized by coalescent granulomas with a complete fibrous encapsulation, extensive central necrosis, and mineralization surrounded by epithelioid macrophages and MNGCs (21). For each LN sample, all granulomas were staged, and a specific stage was given to the sample according to the most represented stage. Pyogranulomas were characterized by a necrotic core with abundant neutrophils surrounded by epithelioid cells and a rim of connective tissue with infiltrate of mononuclear cells. Tissue with normal histological characteristics and no lesion compatible with TBL was considered as negative.

Additional sections from those samples containing TBLs were carried out and stained with Ziehl-Neelsen (ZN) technique for the identification of acid-fast bacilli (AFB). A sample was considered positive for ZN when one or more AFB were detected in at least one high-power field magnification (HPF, 100x) of the sample and, according to the results, the lesions were classified as paucibacillary if it was observed with 1 to 10 AFB bacilli, or pluribacillary if ≥ 11 AFB were observed per HPF (22, 23). Those lesions that yielded a negative result to ZN technique were also subjected to periodic acid-Schiff (PAS) and Gram staining to rule out the presence of fungal structures and bacterial colonies, respectively.

Furthermore, fresh tissue samples from LNs with TBL and a negative result to SIT, real-time PCR from fresh tissue targeting IS*6110*, and culture were subjected to *Mycobacterium avium* subspecies *paratuberculosis* (MAP) detection by using the PCR MAPTB-VK kit (Vacunek S.L., Bizkaia, Spain). DNA template from fresh tissue was run in duplicate for each sample in the MyiQ™2 Two-Color real-time PCR Detection System (Bio-Rad, Hercules, CA, USA) following manufacturer's guidelines. A sample was considered as positive when the Cq value was <40.

### DNA Extraction From Formalin-Fixed Paraffin Embedded (FFPE) Samples

Deoxyribonucleic acid extraction from the paraffin blocks and subsequent real-time PCR were performed from SIT, real-time PCR (from fresh tissue), and bacteriology negative samples, but were positive to the histopathological detection of TBL, independent of their result to ZN technique.

Deoxyribonucleic acid extraction from formalin-fixed paraffine embedded (FFPE) samples was performed using NucleoSpin® DNA FFPE XS kit (Macherey-Nagel, Germany) according to the manufacturer's guidelines with some modifications. In brief, 7 sections of 10 μm thickness were obtained from each tissue block and collected in a sterile microcentrifuge tube of 1.5 ml. A new blade was used for each tissue block to avoid cross contamination. Tissue sections were dewaxed by three incubation steps in xylene at 60°C and 700 rpm for 2 min, followed by two washes with 100% ethanol to remove residual xylene. After dewaxing, tissues were incubated with an open lid at 60°C for 10–12 min to dry the pellet. The tissue was digested in the lysis buffer FL with 20 μl of proteinase K and incubated at 56°C and 700 rpm overnight. Thereafter, Decrosslink Buffer was added and incubated at 90°C and 250 rpm for 30 min, followed by ethanol precipitation. The solution was transferred into a spin column and washed following manufacturer's instructions. DNA was eluted in 60 μl of Elution Buffer. Residual ethanol DNA was removed by incubation at 90°C and 250 rpm for 8 min.

### Real-Time PCR From FFPE Targeting IS*6110*

Deoxyribonucleic acid templates from FFPE samples were diluted up to a final concentration of 150 ng/μl, and amplifications were run in duplicate for each sample in the MyiQ™2 Two-Color real-time PCR Detection System (Bio-Rad, Hercules, CA, USA) following the similar conditions as above mentioned for real-time PCR from fresh tissue.

### Droplet Digital PCR (ddPCR) for Detection of MTC in FFPE Samples

In order to use a technology with a higher Se than real-time PCR for detecting traces of DNA, a droplet digital PCR (ddPCR) targeting IS*611*0 was run in the FFPE samples mentioned above. Indeed, we used the same specific primers and probe targeting a conserved region of IS*6110*. According to Bio-Rad ddPCR system guidelines, each reaction was prepared in a final volume of 20 μl, including 10 μl of ddPCR Supermix for probe (No dUTP), 1.6 μl of IS*6110*-forward (10 mM), 0.8 μl of IS*6110*-reverse (10 mM), 0.6 μl of IS*6110*-probe (FAM-labeled, 10 mM), 4 μl of DNA template, and 3 μl of nuclease-free water. Afterwards, the droplets were generated on the droplet generator according to the manufacturer's instructions. These droplets were carefully transferred to a specific 96-well ddPCR reaction plate (Bio-Rad, Hercules, CA, USA). After heat-sealing, amplifications were run in the C1000 Touch thermal cycler (Bio-Rad, Hercules, CA, USA) under the following cycling conditions: 95°C for 10 min, followed by 40 cycles of 94°C for 30 s and 59°C (annealing/extension) for 1 min, and finally 98°C for 10 min. The temperature ramping rate was set at 2°C/s. Thereafter, the droplets were stored in darkness at 4°C for 12 h. A QX200 Droplet Reader (Bio-Rad, Hercules, CA, USA) was used to read and count the droplets, and the data were then analyzed by using the QuantaSoft Analysis Program (Bio-Rad, Hercules, CA, USA). Samples were disclosed as MTC-positive when the number of positive droplets was ≥ 2. In contrast, samples were MTC-negative if the number of positive droplets was 0, and a droplet number of 0–1 was defined as the ‘gray area’ (24).

### Statistical Analysis

Culture and real-time PCR were considered as gold standard tests for *post-mortem* diagnosis of bTB (5). Thus, the results of the histopathological study were compared with bacteriological culture and real-time PCR from fresh tissue results to estimate diagnostic Se and Sp and positive and negative predictive values (PPV/NPVs) by using WinEpi 2.0 (Faculty of Veterinary, University of Zaragoza, Spain; http://www.winepi.net/uk/index.htm). Cohen's kappa coefficient (κ) analysis was conducted to assess the agreement between two raters. Analysis results were categorized into six categories based on kappa values: no agreement (κ ≤ 0), slight (0.01 < κ ≤ 0.20), weak (0.21 < κ ≤ 0.40), moderate (0.41 < κ ≤ 0.60), good (0.61 < κ ≤ 0.80), and very good (0.81 < κ ≤ 1.00) agreement (WinEpi software 2.0).

## Results

### Bacteriological Culture

Seventy-one LNs yielded a positive result to bacteriological culture. Among these samples, the majority of them corresponded to the tracheobronchial-mediastinal LN (40 out of 71; 56.3%), followed by the retropharyngeal LN (22 out of 71; 31.0%) and, in a lesser extent, the mesenteric LN (9 out of 71; 12.7%) (Table 1). Analyzing the results per animal and considering that an animal was positive when at least one of its 3 LNs resulted positive to bacteriology, 59 out of 230 cattle (25.6%) gave a positive result to bacteriological culture, whereas 171 animals (74.3%) were negative. Eleven animals were positive for more than one LN. From them, 5 cattle were positive for both retropharyngeal and tracheobronchial-mediastinal LNs. Another 5 cattle were positive for tracheobronchial-mediastinal and mesenteric LNs, while only one animal (1 out of 11) was positive for the 3 examined LNs.

**Table 1:** Results of *Mycobacterium tuberculosis* complex (MTC) culture and real-time PCR IS*6110* considering the examined lymph node.

	**Lymph nodes (LN)**	**Positive**	**Negative**	**Total**
		(*n* = 71)	(*n* = 610)	(*n* = 681)
MTC	Retropharyngeal LN	22/71 (31.0%)	205/610 (33.6%)	227/681 (33.3%)
	Tracheobronchial-mediastinal LN	40/71 (56.3%)	186/610 (31.0%)	226/681 (33.2%)
	Mesenteric LN	9/71 (12.7%)	219/610 (35.9%)	228/681 (33.5%)
Real-time PCR IS*6110*		(n = 59)	(n = 622)	(n = 681)
	Retropharyngeal LN	18/59 (30.5%)	209/622 (33.6%)	227/681 (33.3%)
	Tracheobronchial-mediastinal LN	36/59 (61.0%)	190/622 (30.5%)	226/681 (33.2%)
	Mesenteric LN	5/59 (8.5%)	223/622 (35.9%)	228/681 (33.5%)

*LN, Lymph node*.

### Real-Time PCR From Fresh Tissue Targeting IS*6110*

Fifty-nine out of 681 LNs yielded a positive result to real-time PCR from fresh tissue. Among these samples, the majority of them corresponded to the tracheobronchial- mediastinal LN (36 out of 59; 61.1%), followed by the retropharyngeal LN (18 out of 59; 30.5%) and, in a lesser extent, the mesenteric LN (5 out of 59; 8.5%) (Table 1). Analyzing the results per animal and as mentioned above, taking into account that an animal was positive when at least one of its 3 LNs resulted positive to real-time PCR, 59 out of 230 cattle (25.7%) gave a positive result to bacteriological culture, whereas 171 animals (74.3%) were negative. Eleven animals were positive for more than one LN, and from them, seven cattle were positive for both retropharyngeal and tracheobronchial-mediastinal LNs. Another three cattle were also positive for tracheobronchial-mediastinal and mesenteric LNs, while only one animal (1 out of 59) was positive for the 3 examined LNs.

### Serological Analysis

Forty out of 212 sera samples (18.9%) gave a positive result to the ELISA test, while the remaining samples were negative (Table 2). Eighteen ELISA^+^ samples belonged to SIT^+^/MTC^+^/PCR^+^ cattle (18/39; 46.2%), with 19 ELISA^+^ animals corresponding to cattle positive to any of these three techniques (Table 2). Only 3 out of 76 SIT^−^/MTC^−^/PCR^−^ animals (3.9%) presented specific antibodies against *M. bovis*.

**Table 2:** Distribution of ELISA results from the 212 animals according to their intradermal skin test, culture results, and real-time PCR.

	**SIT**^**+**^ **(*****n*** **= 128)**				**SIT**^**−**^**(*****n*** **= 84)**				
	**MTC**^**+**^ **(*****n*** **= 47)**		**MTC**^**−**^**(*****n*** **= 81)**		**MTC**^**+**^ **(*****n*** **= 7)**		**MTC**^**−**^**(*****n*** **= 77)**		**Total**
	**PCR^**+**^**	**PCR^**−**^**	**PCR^**+**^**	**PCR^**−**^**	**PCR^**+**^**	**PCR-**	**PCR+**	**PCR-**	**(*n =* 212)**
**ELISA** ^**+**^	18/39 (46.2%)	2/8 (25.5%)	1/2 (50.0%)	16/79 (20.3%)	0/2 (0.0%)	0/5 (0.0%)	0/1 (0.0%)	3/76 (3.9%)	40/212 (18.9%)
**ELISA** ^**−**^	21/39 (53.8%)	6/8 (75.0%)	1/2 (50.0%) 63/79 (79.7%)		2/2 (100.0%)	5/5 (100.0%)	1/1 (100.0%)	73/76 (96.1%)	172/212 (81.1%)

*SIT, Single Intradermal Tuberculin Test; MTC, Culture of Mycobacterium tuberculosis complex; PCR, real-time PCR of targeting IS6110, (+), Positive; (-), Negative*.

### Histopathological Findings

**Figure 1:**
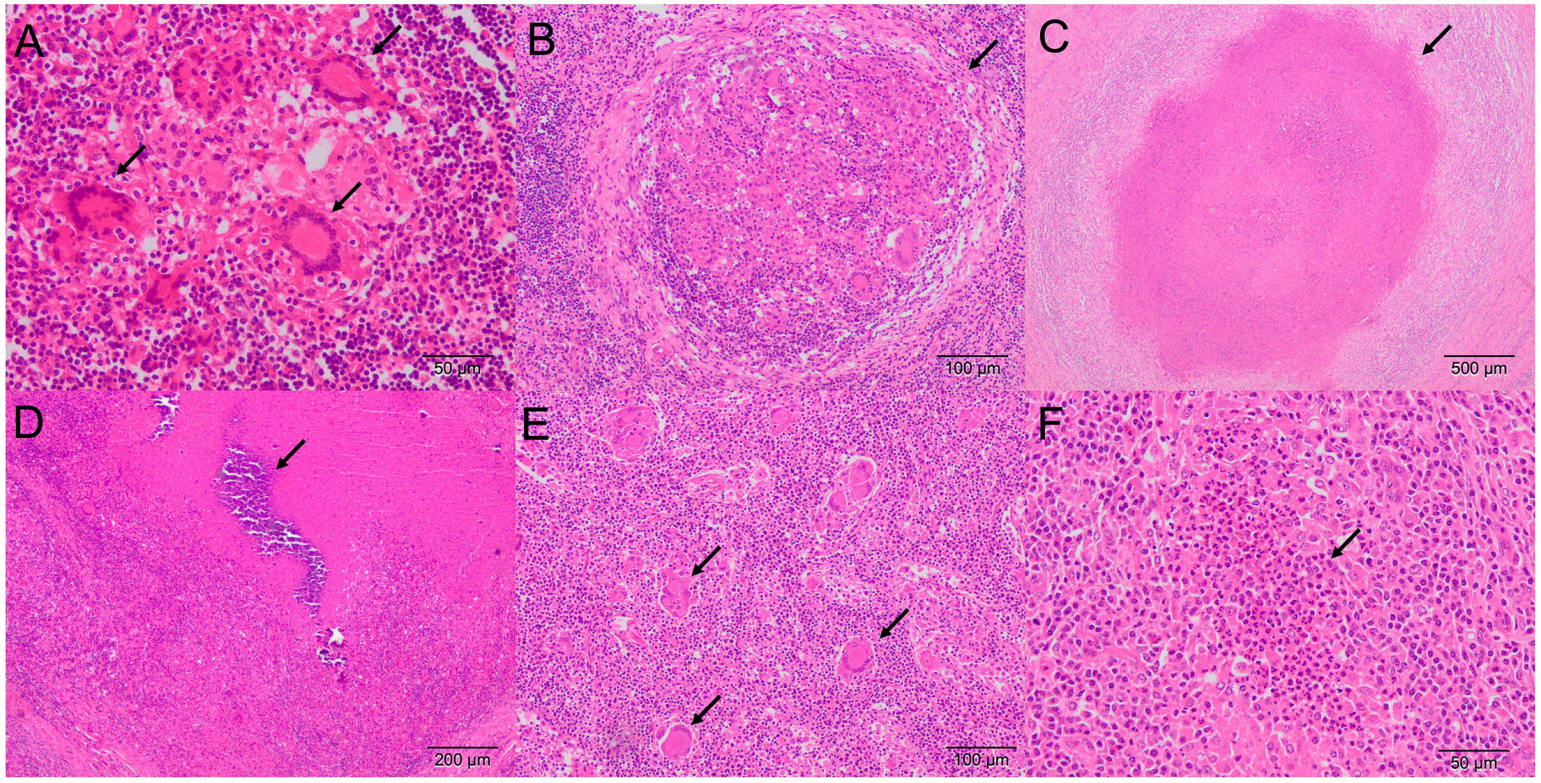
Microscopic lesion of TBLs in LNs (H&E) **(A)** Tracheobronchial-mediastinal LN. Stage I granuloma showing clustered epithelioid macrophages with multinucleated giant cells (MNGCs; arrows) **(B)** Retropharyngeal LN. Stage II granuloma with abundant epithelioid macrophages, lymphocytes, MNGCs, and a fibrous capsule (arrow) **(C)** Mesenteric LN. Stage III granuloma showing a complete fibrous capsule and central necrosis with little mineralization (arrow) **(D)** Tracheobronchial-mediastinal LN. Stage IV coalescent granuloma with a complete fibrous encapsulation, extensive central necrosis, and mineralization (arrow) **(E)** Mesenteric LN. Multiple scattered Langhans-type MNGCs (arrows). **(F)** Retropharyngeal LN. Pyogranuloma with polymorphonuclear cells (arrow), apoptotic bodies, and abundant necrotic center.

**Figure 2:**
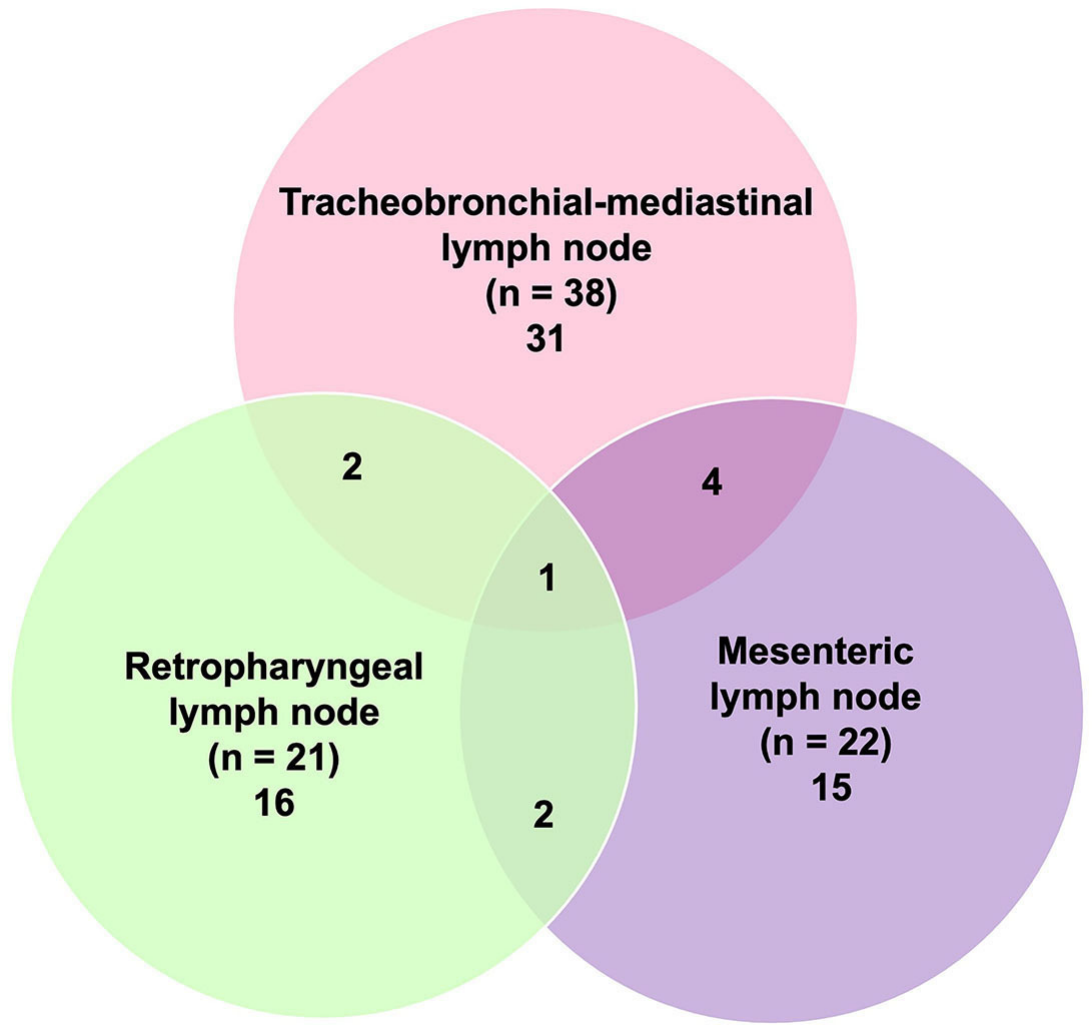
Distribution of tuberculosis-like lesions (TBLs) (*n* = 81) according to the affected lymph node (LN).

Tuberculosis-like lesions were observed in 81 out of 681 (11.9%) examined LNs and were distributed as TB granuloma in 60 samples (60 out of 81; 74.1%), pyogranuloma in 5 samples (5 out of 81; 6.2%), and scattered Langhans-type MNGCs in 16 samples (16 out of 81; 19.7%) (Figure 1, Table 3). TB granulomas were highly represented in the tracheobronchial-mediastinal LN, followed by the retropharyngeal LN (33 out of 38, 86.8%; and 16 out of 21, 76.2%, respectively). The presence of scattered Langhans-type MNGCs was another type of TBL frequently observed in the mesenteric LN (9 out of 22; 40.9%). Seventy one out of 230 cattle (30.9%) presented TBL. Nine animals were positive for more than one LN, and from them, 2 cattle were positive for both retropharyngeal and tracheobronchial-mediastinal LNs and tracheobronchial-mediastinal and mesenteric LNs. Another 4 cattle were positive for tracheobronchial-mediastinal and mesenteric LNs, while only one animal (1 out of 9) was positive for the 3 examined LNs (Figure 2).

**Table 3:** Distribution of microscopic lesions in different lymph nodes.

	**TBL (*****n*** **= 81; 81/681; 11.9%)**					
**Histopathological lesions**	**TB Granuloma** **(*n* = 60)**	**Pyogranuloma** **(*n* = 5)**	**Giant Cell** **(*n* = 16)**	**Other lesions** **(*n* = 17)**	**No lesion** **(*n* = 583)**	**Total** **(*n* = 681)**
Retropharyngeal LN	16/81 (19.7%)	2/81 (2.5%)	3/81 (3.7%)	3/17 (17.6%)	203/583 (34.8%)	227
Tracheobronchial-mediastinal LN	33/81 (40.7%)	1/81 (1.2%)	4/81 (4.9%)	1/17 (5.9%)	187/583 (32.1%)	226
Mesenteric LN	11/81 (13.6%)	2/81 (2.5%)	9/81 (11.1%)	13/17 (76.5%)	193/583 (33.1%)	228

*TBL, Tuberculosis-like lesions; TB, tuberculous; LN, Lymph node*.

**Figure 3:**
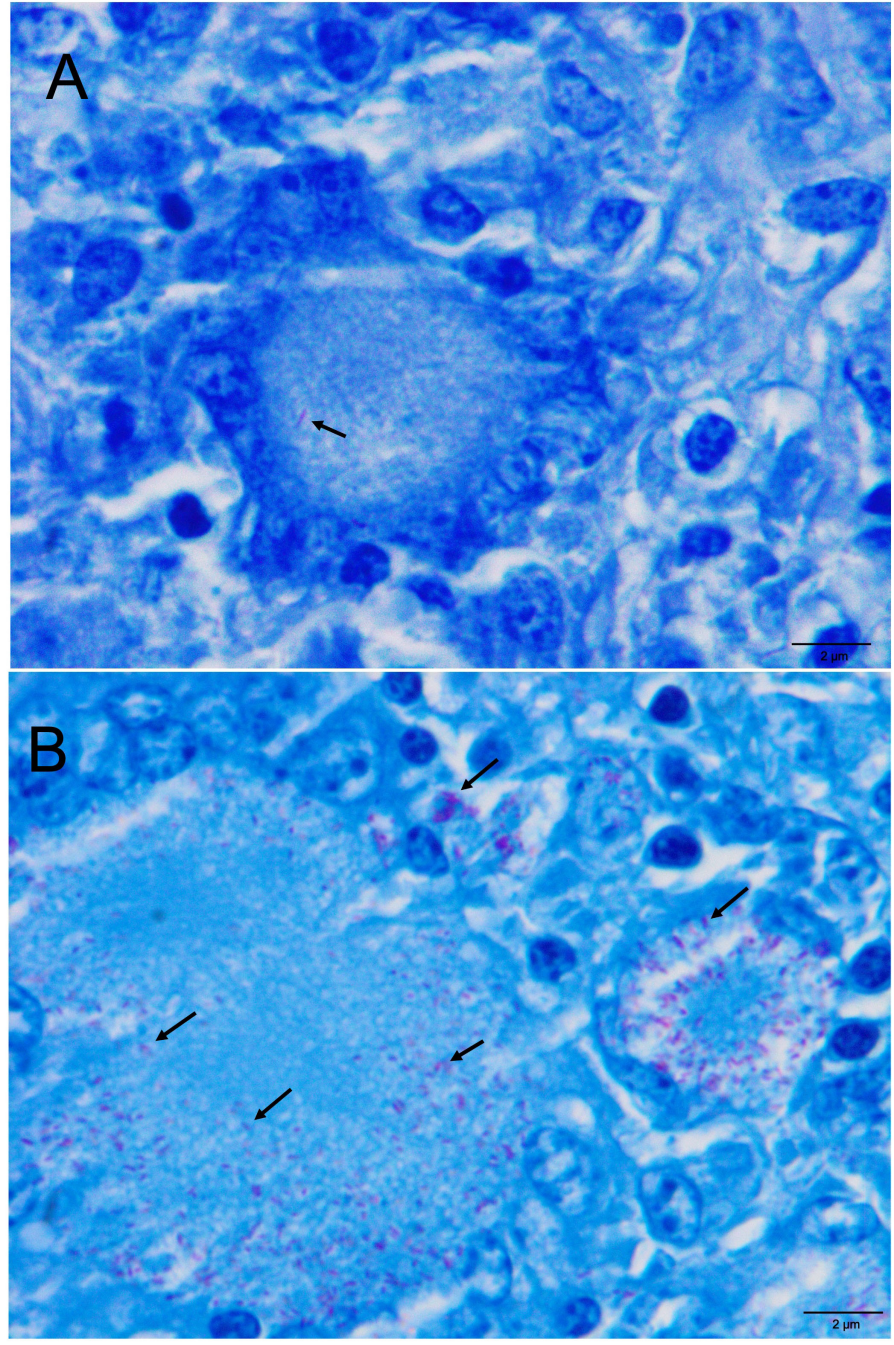
**(A)** Retropharyngeal LN. Paucibacillary lesion. The arrowhead shows acid fast bacilli (AFB) within cytoplasm of a Langhans-type MNGCs (Ziehl-Neelsen, ZN) **(B)**. Mesenteric LN. Pluribacillary lesion. Numerous AFB within the cytoplasm of Langhans-type MNGCs and epithelioid cells (ZN).

Ziehl-Neelsen technique was performed to determine the presence of AFB in samples with TBL (Table 4). Sixty-one out of 81 samples (75.3%) with TBL yielded a positive result to ZN technique, mainly corresponding to TB granuloma (54 out of 81; 66.7%). Only 6 out of 16 samples with Langhans-type MNGCs presented a ZN^+^ result (37.5%), and just 1 sample out of 5 (20.0%) with pyogranuloma. As depicted in Table 5, from all ZN^+^ LNs, 43 showed a paucibacillary form (Figure 3A) (22 in tracheobronchial-mediastinal LN, 15 in retropharyngeal LN and 6 in mesenteric LN), whereas 18 showed a pluribacillary form (Figure 3B) (8 in mesenteric LN, 7 in tracheobronchial-mediastinal LN, and 3 in retropharyngeal LN). Nineteen out of the 81 TBL samples were positive for ZN (13 paucibacillary and 8 pluribacillary) and negative for SIT, MTC, and PCR. Neither bacterial colonies by H&E and Gram staining nor fungal structures by PAS staining were evidenced in ZN^−^ samples.

**Table 4:** Relationship between microscopic lesions and the presence of acid-fast bacilli (AFB) in different lymph nodes.

	**TBL (*****n*** **= 81; 100%)**						
	**TB Granuloma** **(*****n*** **= 60; 74.1%)**		**Giant cells** **(*****n*** **= 16; 19.7%)**		**Pyogranuloma** **(*****n*** **= 5; 6.2%)**		**Total**
	**ZN^+^ (*n* = 54)**	**ZN^−^ (*n* = 6)**	**ZN^+^ (*n* = 6)**	**ZN^−^ (*n* = 10)**	**ZN^+^ (*n* = 1)**	**ZN^−^ (*n* = 4)**	**(*n* = 81)**
Retropharyngeal LN	16/54 (26.7%)	0/6 (0.0%)	1/6 (6.2%)	2/10 (12.5%)	1/1 (20.0%)	1/4 (20.0%)	21/81
Tracheobronchial-mediastinal LN	29/54 (48.3%)	4/6 (6.7%)	0/6 (0.0%)	4/10 (25.0%)	0/1 (0.0%)	1/4 (20.0%)	38/81
Mesenteric LN	9/54 (15.0%)	2/6 (3.3%)	5/6 (31.2%)	4/10 (25.0%)	0/1 (0.0%)	2/4 (40.0%)	22/81

*TBL, Tuberculosis-like lesions; ZN, Ziehl-Neelsen; LN, Lymph node*.

**Table 5:** Classification of the lesions according to the Ziehl-Neelsen (ZN) staining and the number of AFB, paucibacillary or pluribacillary, in the different lymph nodes.

	**ZN**^**+**^ **(*****n*** **= 61; 75.3%)**		**ZN^**−**^(*n* = 20; 24.7%)**	**Total**
	**Paucibacillary**	**Pluribacillary**		**(*n* = 81)**
Retropharyngeal LN	15/43 (24.6%)	3/18 (4.9%)	3/20 (15.0%)	21
Tracheobronchial-mediastinal LN	22/43 (46.6%)	7/18 (4.9%)	9/20 (45.0%)	38
Mesenteric LN	6/43 (11.5%)	8/18 (11.5%)	8/20 (40.0%)	22

*LN, Lymph node; ZN, Ziehl-Neelsen; (+), Positive; (−), Negative*.

Tuberculosis-like lesions were identified in a total of 61 cattle, with 53 animals presenting a positive result to SIT, MTC, and/or PCR. Noteworthy, TBL were identified in all animals with a positive result to bacteriology or to PCR, independently of the SIT result, except in 15 out of 59 MTC^+^ animals and 6 out of 48 PCR^+^ animals in which histopathological lesions were not observed (Table 6). Interestingly, when the histopathology was compared to SIT and bacteriological culture results per animal, 18 out of 83 SIT^−^, MTC^−^, and PCR^−^ animals presented TBL. Among them, 8 showed TB granuloma, 1 pyogranuloma, and 9 Langhans-type MNGCs. In these animals, ZN results evidenced the presence of AFB in 5 out of 8 TB granuloma and 6 out of 9 Langhans-type MNCGs_._ The pyogranuloma was negative for ZN. As mentioned above, neither bacterial colonies nor fungal structures were evidenced by H&E, Gram, and PAS staining. Remarkably, MAP was detected in 6 of these samples by real-time PCR, all of them belonging to mesenteric LNs with pluribacillary ZN staining. Among the animals which presented other types of lesions other than TBL, 2 of them presented parasitic migration, 1 choristoma, 10 psammoma bodies, and 2 with inflammatory infiltration.

**Table 6:** Distribution of the results collected from the 230 animals which were positive or negative to single intradermal skin test and subclassified by culture of MTC and real-time PCR targeting IS*6110 vs*. histopathological lesions.

	**SIT**^**+**^ **(*****n*** **= 139)**				**SIT**^**−**^**(*****n*** **= 91)**			
**Histopathological**	**MTC**^**+**^ **(*****n*** **= 51)**		**MTC**^**−**^**(*****n*** **= 88)**		**MTC**^**+**^ **(*****n*** **= 8)**		**MTC**^**−**^**(*****n*** **= 83)**	
**lesions**	**PCR^**+**^**	**PCR^**−**^**	**PCR^**+**^**	**PCR^**−**^**	**PCR^**+**^**	**PCR^**−**^**	**PCR^**+**^**	**PCR^**−**^**
TB Granuloma	40/43 (93.0%)	0/8 (0.0%)	1/2 (50.0%)	3/86 (34.9%)	0/2 (0.0%)	1/6 (1.7%)	0/1 (0.0%)	8/82 (9.8%)
Giant cell	0/43 (0.0%)	0/8 (0.0%)	0/2 (0.0%)	4/86 (4.6%)	0/2 (0.0%)	2/6 (3.3%)	0/1 (0.0%)	9/82 (10.9%)
Pyogranuloma	1/43 (2.3%)	0/8 (0.0%)	0/2 (0.0%)	1/86 (1,7%)	0/2 (0.0%)	0/6 (0.0%)	0/1 (0.0%)	1/82 (1.2%)
Other lesion	0/43 (0.0%)	0/8 (0.0%)	0/2 (0.0%)	11/86 (12.8%)	0/2 (0.0%)	0/6 (0.0%)	0/1 (0.0%)	4/82 (4.9%)
No lesion	2/43 (4.7%)	8/8 (0.0%)	1/2 (50.0%)	67/86 (77.9%)	2/2 (100.0%)	3/6 (50.0%)	1/1 (100.0%)	60/82 (73.2%)

*TB, Tuberculous; SIT, Single intradermal Tuberculin Test; MTC, Culture of Mycobacterium tuberculosis complex; PCR, real-time PCR of targeting IS6110; (+), Positive; (−), Negative*.

Furthermore, the relationship between ZN staining (paucibacillar vs. pluribacillar patterns) and the stage of TB granuloma with respect to the result to the different techniques (SIT, ELISA, bacteriology, and real-time PCR) was also analyzed. A total of 54 TB granulomas with a ZN result were included in this study. These samples corresponded to 40 granulomas with a paucibacillar ZN staining and 14 granulomas with a pluribacillar staining. Paucibacillary granulomas were distributed as follows: 1 stage I granuloma, 2 stage II granulomas, 8 stage III granulomas, and 29 stage IV granulomas. Pluribacillary granulomas consisted of 6 stage I granulomas, 5 stage III granulomas, and 3 stage IV granulomas. No stage II granuloma was found.

According to this distribution, paucibacillar stage IV granulomas prevailed in our study and corresponded to cattle positive to at least two of the analyzed techniques: 10 SIT^+^/ELISA^+^/MTC^+^/PCR^+^ animals; 1 SIT^+^/ELISA^+^/MTC^−^/PCR^+^ animal; 1 SIT^+^/ELISA^+^/MTC^−^/PCR^−^ animal; 10 SIT^+^/ELISA^−^/MTC^+^/PCR^+^ animals; and 3 SIT^+^/MTC^+^/PCR^+^ animals. Two stage IV granulomas yielded a negative result to SIT, bacteriology, and PCR (no serum was available for ELISA). In this line, paucibacillary stage III granulomas belonged to cattle with positive result to at least three of the analyzed techniques, namely, 3 SIT^+^/ELISA^+^/MTC^+^/PCR^+^ animals, 4 SIT^+^/ELISA^−^/MTC^+^/PCR^+^ animals, and 1 SIT^+^/MTC^+^/PCR^+^ animal. The low number of animals in which stage I and stage II granulomas prevailed did not allow analyzing their relationship with regards to the other techniques.

Pluribacillar granulomas, independently of their stage, corresponded to animals positive to at least three of the analyzed techniques, with one stage I and one stage IV granuloma being positive only to SIT. Four stage I granulomas belonged to animals negative to SIT, bacteriology, and PCR (no serum was available for ELISA).

### Estimates of Sensitivity and Specificity and Agreement of Histopathology With Respect to the Reference Techniques

Table 7 shows the results of Se, Sp, and κ. Estimates of Se and Sp were calculated for SIT, ELISA, and histopathology with respect to the bacteriological culture and real-time PCR IS*6110* from fresh tissues.

**Table 7:** Estimates of sensitivity and specificity of the diagnostic techniques compared to against MTC culture as gold standard either alone or in parallel combination together with real-time PCR targeting IS*6110*.

		**Sensitivity (Se)**		**Specificity (Sp)**			
	**Diagnostic test**	**%**	**CI_**95**_%**	**%**	**CI_**95**_%**	**κ**	**Agreement**
MTC	SIT	86.4	(77.7–95.2)	48.5	(41.0–56.0)	0.242	Weak
	ELISA	37	(24.2–49.9)	87.3	(82.2–92.5)	0.256	Weak
	Histopathology (H&E)	74.6	(63.5–85.7)	84.2	(78.7–89.7)	0.551	Moderate
Real time PCR IS6110	SIT	93.8	(86.9–100.6)	48.4	(41.1–55.6)	0.248	Weak
	ELISA	43.2	(28.5–57.8)	87.5	(82.5–92.5)	0.317	Weak
	Histopathology (H&E)	87.5	(78.1–96.9)	84.1	(78.7–89.4)	0.608	Good

*SIT, Single intradermal tuberculin test; H&E, Hematoxylin and eosin staining; MTC, Mycobacterium tuberculosis Complex*.

Single intradermal tuberculin test results presented good Se (86.4%; CI_95_: 77.7-95.2%) but low Sp (48.5%; CI_95_: 41.0-56.0%) compared with bacteriological culture. The PPV and NPV were 36.7% (CI_95_: 28.7-44.7%) and 91.2% (CI_95_: 85.4-97.0%), respectively. When compared with real-time PCR, the Se and Sp of SIT were 93.8% and 48.4%, respectively, and the PPV and NPV were 32.4% (CI_95_: 24.6-40.2%) and 96.7% (CI_95_: 93.0-100.4%), with a weak agreement (κ = 0.248) among techniques.

Enzyme-linked immunosorbent assay results presented low Se (37%; CI_95_: 24.2-49.9%) but good Sp (87%; CI_95_: 82.2-92.5%). The PPV and NPV were 50% (CI_95_: 34.5-65.5%) and 80.2% (CI_95_: 74.3-86.1%), respectively. When compared with real-time PCR from fresh tissue, the Se and Sp of ELISA were 43.2% and 87.5%, respectively, and the PPV and NPV were 47.5% (CI_95_: 32.0-63.0%) and 85.5% (CI_95_: 80.2-90.7%), with a weak agreement (κ = 0.317) among techniques.

Regarding histopathology, it presented an acceptable Se (74.6%; CI_95_: 63.5-85.7%) and Sp (84.2%; CI_95_: 78.7-89.7%). The PPV and NPV were 62.0% (CI_95_: 50.7-73.3%) and 90.6% (CI_95_: 86.0-95.1%), respectively, with a moderate agreement (κ = 0.551). On the other hand, the comparison of histopathology with the results of real-time PCR showed a Se of 87.5% (CI_95_ 78.1-96.9%) and a Sp of 84.1% (CI_95_ 78.7–89.4%) with PPV and NPV of 59.2% (CI_95_: 47.7-70.6%) and 96.2% (CI_95_: 93.3-99.2%), respectively. A good agreement (κ = 0.608) was observed among techniques.

### Real-Time PCR From FFPE Targeting IS*6110*

Eighteen out of 83 SIT^−^, MTC^−^, and PCR^−^ (from fresh tissue) animals presented TBL and were subsequently subjected to real-time PCR analysis directly from the paraffin blocks. One sample was not available for the study because the paraffin block was already exhausted. One sample out of seventeen (5.9%) was positive by means of real-time PCR targeting IS*6110*. The IAC amplified in all samples without partial inhibition. The real-time PCR-positive result belonged to a tracheobronchial-mediastinal LN in a sample that showed TB granuloma in histopathology as TBL and paucibacillary lesion in ZN technique. This animal only presented lesion in this LN.

### Droplet Digital PCR (ddPCR) Targeting IS6110

**Figure 4:**
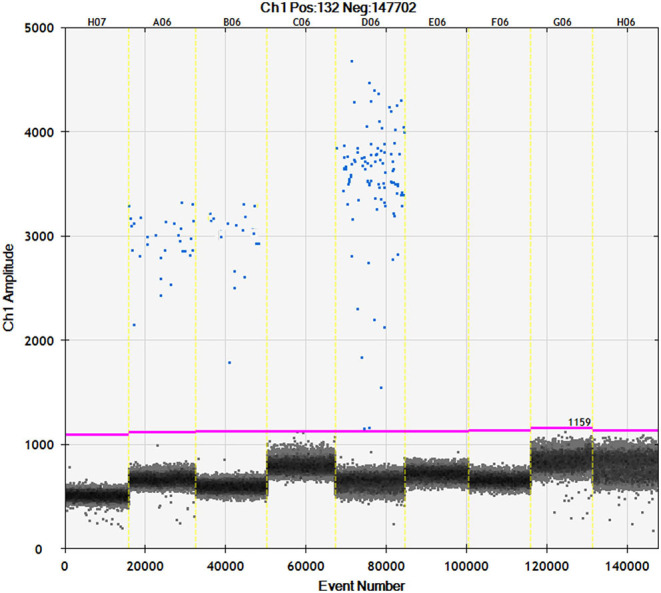
Channel 1 results for droplet separation based on simplex assays. Droplet separation based the IS*6110* simplex assay results. Positive droplets were observed at an amplitude of about 3,000 while negative droplets were observed at an amplitude of about 1,100.

In addition, ddPCR was carried out from paraffin blocks of the 17 SIT^−^, MTC^−^, and PCR^−^ samples with TBL. Only 2 out of 17 samples (11.7%) were positive by ddPCR targeting IS*6110*, with one of them coinciding with the sample positive to real-time PCR from FFPE (Figure 4). The other ddPCR^+^ result belonged to a tracheobronchial-mediastinal LN that showed MNGCs in histopathology to be TBL and had a negative result to ZN technique, without lesion in any other LN in the same animal. No sample was observed in the “gray area.”

## Discussion

Improving the diagnosis of bTB in cattle is a cornerstone in control and eradication programs against this disease, being fundamental in the rapid and effective identification of infected animals. Current diagnostic tests for bTB present a low to moderate Se, therefore, the combination of different diagnostic tests, both *ante-mortem* and *post-mortem*, represents a smart approach to improve the diagnosis against MTC. Histopathology is an early, rapid, and economic technique that allows us to identify and grade microscopic lesions associated with MTC infection. In addition, it visualizes the presence of mycobacteria within these lesions by additional techniques (ZN staining). Hence, its combination with reference techniques, such as bacteriological culture and real-time PCR, deserves more attention. In the present study, 212 sera samples and 681 LNs (227 retropharyngeal, 226 tracheobronchial-mediastinal and 228 mesenteric LNs) were collected from 230 slaughtered cattle from the national bTB control and eradication program. A higher number of culture positive samples and TBL were obtained from the tracheobronchial-mediastinal LN, followed by the retropharyngeal LN. These results are in agreement with the exposure of cattle to contaminated aerosols as the main route of entry of *M. bovis* (8, 9, 25). Interestingly, one cattle presented culture positive and PCR positive results together with TBL only in the mesenteric LN, which suggests the ingestion of food and/or water contaminated with *M. bovis* (9, 25–28). Although a systematic sampling of LNs is included in the bTB eradication programs (7), the veterinary inspection and sampling at the slaughterhouse is usually limited to head and thoracic cavity LNs from a logistic perspective due to the speed of the slaughter chain. This is one of the main limitations of the current study due to the lack of a more systematic sampling of LNs. However, our results highlight the importance of including mesenteric LNs in the routine sampling to avoid false negative animals limiting the persistence and spread of mycobacterium in negative herds.

Enzyme-linked immunosorbent assay has been considered for *ante-mortem* diagnosis with its simplicity as main advantage, but presenting a low Se (64.6%), which is primarily associated with a delayed development of humoral immunity (around 3–12 weeks) (9). In our study, from the total of MTC^+^ animals, approximately one third were ELISA^+^ (20 out of 54; 37.0%), which led to a low Se. The use of ELISA for bTB diagnosis has been argued by many authors for its utility to detect anergic animals (9). In our case, only 3 out of 77 animals (5.9%) were ELISA^+^ and SIT^−^/MTC^−^/PCR^−^. However, these animals did not present TBL. In addition, 15 out of 76 SIT^−^/MTC^−^/PCR^−^ animals presented TBL, but disclosed a negative result to ELISA, pointing out that the contribution of the ELISA test for bTB diagnosis is not significant in the conditions of the present study.

Histopathology allows the identification of lesions compatible with TBL and lesions other than those caused by mycobacteria. The predominant TBL observed in our study was the TB granuloma. It is noteworthy that other TBL, such as pyogranuloma or Langhans-type MNGCs, were observed. Pyogranulomas may be caused by the concomitant infection of mycobacteria and pyogenic bacteria, such as *Streptococcus* spp. or *Trueperella pyogenes* (29, 30). MNGCs are grossly unnoticed and, interestingly, in our study, were observed in 16 out of 81 LNs of cattle with TBL, being observed more frequently in the mesenteric LNs (9 out of 81; 11.1%). Six out of these 9 mesenteric LNs with Langhans-type MNGCs belonged to SIT^−^/MTC^−^/PCR^−^ cattle and presented a positive result to MAP PCR and a pluribacillar (5 LNs) or paucibacillar (1 LN) pattern with ZN staining. Therefore, these cases were consistent with field cases of paratuberculosis, not with an infection by MTC, and emphasize the interest of performing a differential diagnosis when monitoring lesions in LNs from the digestive system (31). Other lesions, including parasitic migration, hemosiderosis, and psammoma bodies, among others, were also observed. Frequently, reactive LNs cannot be grossly differentiated with certainty into those with or without TBL (5, 13, 30). Besides the use of histopathology to confirm the microscopic diagnosis of a TBL, the presence of AFB can also be evidenced through the ZN technique as stated above. Usually, paucibacillary lesions are observed in cattle, however, AFB may not be detected although *M. bovis* is isolated in the bacteriological culture from the same sample (9, 11, 21). ZN^+^ mycobacteria are frequently observed in giant cells or in areas of necrosis (32, 33), with the presence of at least one mycobacterium being enough to consider a result as positive. In the present study, ZN technique yielded positive results in a similar percentage (61/81; 75.3%) as described by others (33), with most of the samples preferably presenting paucibacillary lesions (48/61), confirming the usefulness of this technique to confirm the involvement of mycobacteria within a concrete lesion.

Our results showed that TB granuloma was the predominant lesion within TBL (60/81; 74.1%), identifying in most cases AFB by ZN technique (54 out of 60), which highlights a high association between TB granuloma and the presence of AFB. Forty out of 43 animals showing TB granuloma belonged to SIT^+^/MTC^+^/PCR^+^ cattle. In addition, three out of 86 SIT^+^ and MTC^−^ and PCR^−^ animals also presented TB granuloma as a TBL, pointing out the interest of the histopathology to confirm the diagnosis in these cases, more importantly in SIT^+^ cases to allow a faster confirmation of bTB diagnosis. Interestingly, 8 SIT^−^/MTC^−^/PCR^−^ animals presented TB granuloma, with 5 of them disclosing a positive result to ZN but negative to ELISA, evidencing the capability of this technique to identify infected animals negative to the reference techniques. Indeed, real-time PCR and ddPCR from FFPE tissues were able to amplify MTC from one of these TB granulomas, which presented a paucibacillary ZN staining, supporting this statement. The implication of a foreign body reaction, bacteria or fungi was ruled out by the histopathological characteristics of the lesion, Gram, and PAS staining, respectively, along with the participation of MAP by real-time PCR. Therefore, these animals may correspond with anergic and/or immunosuppressed animals, with a low bacterial load, corroborating the role of the histopathological study to identify TBL in a rapid fashion (approximately 48 h), allowing an early diagnostic approach. Nonetheless, a more complete differential diagnosis would allow discarding the involvement of any other potential microorganism in these lesions.

Regarding pyogranulomas, only 1 out of 5 was found to be ZN^+^, characterized by a pluribacillary lesion and corresponding with an MTC^+^ and PCR^+^ animal. On the other hand, one SIT^−^/MTC^−^/PCR^−^ animal presented a TBL consisting with a pyogranuloma. However, this lesion was ZN^−^ and the animal also yielded a negative result to ELISA, indicating that it might be a disease other than tuberculosis (23).

Analyzing the stage of the granuloma and the ZN pattern with respect to the different methods analyzed in our study, paucibacillar stage IV granulomas were overrepresented with respect to other stages or pluribacillar granulomas. This result highlights the advanced stages of the infection observed in our study, corresponding probably with around 6 months post-infection or later according to previous results (34). Our results do not draw a clear association of granuloma stage or pauci-/pluri-bacillary pattern and the results to the different techniques since most of the animals with different stages of granuloma yielded a positive result to SIT, bacteriological culture, and PCR. Only ELISA results presented a random distribution independently of the stage of the granuloma and ZN pattern. However, it is of interest to remark that in some cases, although in a low proportion, paucibacillar stage IV or pluribacillar stage I granulomas were detected in cattle, negative to all the techniques under study. The number of animals with TB granuloma included in our study together with the distribution of animals according to the stage of the granuloma and ZN pattern did not allow us to examine the relationship between the number of AFB and the results to the different diagnostic methods

Single intradermal tuberculin test-positive/MTC^−^/PCR^−^/TBL^+^ animals (8 out of 86; 9.3%) may be associated with several factors, such as a very low bacterial load, the encapsulation of the granulomas (35), the decontamination process of the microbiological culture (8), or as suggested by Cassidy and co-authors (31), with animals that have eliminated the infection but have been sensitized to the disease. Although co-infection with other pathogens or exposure to other mycobacterial species, including members of the *M. avium* complex (MAP) and environmental mycobacteria, can affect SIT test performance and generate SIT false positives (5, 10, 36), the evidence of TBL together with ZN positive results allow confirming SIT positivity in our case. Nonetheless, according to our results, special caution should be taken when evaluating mesenteric LNs with Langhans-type MNGCs as the sole lesion.

According to the inherent difficulties described above for each one of these techniques to detect bTB in cattle when used in single, the diagnostic performance of SIT, ELISA, and histopathology compared with respect to bacteriology displayed a low to moderate Se and Sp and a weak to moderate agreement among tests. However, when histopathology was compared with real-time PCR from fresh tissues, it presented a higher Se and a good agreement among both techniques (κ = 0.608), evidencing the suitability of including histopathology as a complimentary diagnostic tool which may help improving bTB diagnosis and disease control.

## Conclusions

Our results confirm that histopathology is a valuable diagnostic tool with an acceptable Se (87.5%) and Sp (84.1%) to be used alone or together with SIT, real-time PCR, and bacteriological culture. Furthermore, histopathology allows not only a rapid confirmation of SIT, PCR, and culture positive results, but also detecting positive animals that yield negative results to these techniques. For these reasons, this tool should be systematically included in bTB surveillance and eradication programs.

## Data Availability Statement

The raw data supporting the conclusions of this article will be made available by the authors, without undue reservation.

## Ethics Statement

Ethical review and approval was not required for the animal study because no purpose killing of animals was performed for this study, so no ethical or farmer's consent approval was required.

## Author Contributions

JG-L, LC, IL, and CT conceived, designed, and performed the project. JS-C, AG-R, IR-T, EV-S, and FL-M helped in the sample collection. JS-C, AG-R, and LG-G made the laboratory experiments. AM helped in statistical analysis. FL-M wrote the manuscript. IR-T and JG-L reviewed the manuscript. LC and JG-L contributed to the reagents, materials, and analysis tools. All authors contributed to the article and approved the submitted version.

## Funding

This work was supported by the research project New Measures and Techniques to Control Bovine Tuberculosis in Andalusia (Financial support for Operational Groups of the European Innovation Partnership for Agricultural Productivity and Sustainability (EIP-AGRI) (GOP2I-CO-16-0010). FL-M was supported by a doctoral grant from ANID (National Research and Development Agency) (Doctoral grant Chile/2019/72200324).

## Conflict of Interest

The authors declare that the research was conducted in the absence of any commercial or financial relationships that could be construed as a potential conflict of interest.

## Publisher's Note

All claims expressed in this article are solely those of the authors and do not necessarily represent those of their affiliated organizations, or those of the publisher, the editors and the reviewers. Any product that may be evaluated in this article, or claim that may be made by its manufacturer, is not guaranteed or endorsed by the publisher.

## References

[1] Rodriguez-Campos S Smith NH Boniotti MB Aranaz A. Overview and phylogeny of Mycobacterium tuberculosis complex organisms: implications for diagnostics and legislation of bovine tuberculosis. Res Vet Sci. (2014) 97:S5–19. 10.1016/j.rvsc.2014.02.009 24630673

[2] Mohamed A. Bovine tuberculosis at the human–livestock–wildlife interface and its control through one health approach in the Ethiopian Somali Pastoralists: a review. One Heal. (2020) 9:100113. 10.1016/j.onehlt.2019.100113 31872034 PMC6911947

[3] OIE. World Organisation for Animal Health. Infection with mycobacterium tuberculosis complex. In Autor, editor. Terrestrial Animal Health Code. 28th ed. Paris: World Organisation for Animal Health (2019). p. 1–6.

[4] Adams LG. *In vivo* and *in vitro* diagnosis of Mycobacterium bovis infection. OIE Rev Sci Tech. (2001) 20:304–24. 10.20506/rst.20.1.1267 11288518

[5] de la Rua-Domenech R Goodchild AT Vordermeier HM Hewinson RG Christiansen KH Clifton-Hadley RS. Ante mortem diagnosis of tuberculosis in cattle: a review of the tuberculin tests, γ-interferon assay and other ancillary diagnostic techniques. Res Vet Sci. (2006) 81:190–210. 10.1016/j.rvsc.2005.11.005 16513150

[6] Reviriego Gordejo FJ Vermeersch JP. Towards eradication of bovine tuberculosis in the European Union. Vet Microbiol. (2006) 112:101–9. 10.1016/j.vetmic.2005.11.034 16388921

[7] EU. REGLAMENTO DELEGADO (UE) 2020/689 DE LA COMISIÓN de 17 de diciembre de 2019 por por el que se completa el Reglamento (UE) 2016/429 del Parlamento Europeo y del Consejo en lo referente a las normas de vigilancia, los programas de erradicación y el estatus. D Of la Unión Eur (2020) 2019

[8] Corner LAL Gormley E Pfeiffer DU. Primary isolation of Mycobacterium bovis from bovine tissues: conditions for maximising the number of positive cultures. Vet Microbiol. (2012) 156:162–71. 10.1016/j.vetmic.2011.10.016 22074859

[9] Ramos DF Silva PEA Dellagostin OA. Diagnosis of bovine tuberculosis: review of main techniques. Braz J Biol. (2015) 75:830–7. 10.1590/1519-6984.23613 26675901

[10] Byrne AW Graham J Brown C Donaghy A Guelbenzu-Gonzalo M McNair J. Modelling the variation in skin-test tuberculin reactions, post-mortem lesion counts and case pathology in tuberculosis-exposed cattle: effects of animal characteristics, histories and co-infection. Transbound Emerg Dis. (2018) 65:844–58. 10.1111/tbed.12814 29363285

[11] Schiller I Oesch B Vordermeier HM Palmer MV Harris BN Orloski KA. Bovine tuberculosis: a review of current and emerging diagnostic techniques in view of their relevance for disease control and eradication. Transbound Emerg Dis. (2010) 57:205–20. 10.1111/j.1865-1682.2010.01148.x 20561288

[12] Nuñez-Garcia J Downs SH Parry JE Abernethy DA Broughan JM Cameron AR. Meta-analyses of the sensitivity and specificity of ante-mortem and post-mortem diagnostic tests for bovine tuberculosis in the UK and Ireland. Prev Vet Med. (2018) 153:94–107. 10.1016/j.prevetmed.2017.02.017 28347519

[13] Bezos J Casal C Romero B Schroeder B Hardegger R Raeber AJ. Current ante-mortem techniques for diagnosis of bovine tuberculosis. Res Vet Sci. (2014) 97:S44–52. 10.1016/j.rvsc.2014.04.002 24768355

[14] Fontana S Pacciarini M Boifava M Pellesi R Casto B Gastaldelli M. Development and evaluation of two multi-antigen serological assays for the diagnosis of bovine tuberculosis in cattle. J Microbiol Methods. (2018) 153:118–26. 10.1016/j.mimet.2018.09.013 30248441

[15] Courcoul A Moyen JL Brugère L Faye S Hénault S et al. Estimation of sensitivity and specificity of bacteriology, histopathology and PCR for the confirmatory diagnosis of bovine tuberculosis using latent class analysis. PLoS ONE. (2014) 9: 0090334. 10.1371/journal.pone.0090334 24625670 PMC3953111

[16] Lorente-Leal V Liandris E Castellanos E Bezos J Domínguez L de Juan L. Validation of a real-time PCR for the detection of mycobacterium tuberculosis complex members in Bovine tissue samples. Front Vet Sci. (2019) 6:1–9. 10.3389/fvets.2019.00061 30886855 PMC6409304

[17] Sánchez-Carvajal JM Galán-Relaño Á Ruedas-Torres I Jurado-Martos F Larenas-Muñoz et al. Real-Time PCR validation for *Mycobacterium tuberculosis complex detection targeting IS 6110* directly from bovine lymph nodes. Front in Vet Sci. (2021) 8:643111. 10.3389/fvets.2021.643111 33981742 PMC8109245

[18] España. Real Decreto 2611/1996, de 20 de diciembre, por el que se regulan los programas nacionales de erradicación de enfermedades de los animales. Bol Of del Estado (1996) 21 diciembre 1996 1–37. (19)

[19] Thierry D Brisson-Noel A Vincent-Levy-Frebault V Nguyen S Guesdon JL Gicquel B. Characterization of a Mycobacterium tuberculosis insertion sequence, IS6110, and its application in diagnosis. J Clin Microbiol. (1990) 28:2668–73. 10.1128/jcm.28.12.2668-2673.1990 2177747 PMC268253

[20] Michelet L de Cruz K Karoui C Tambosco J Moyen JL Hénault S. Second line molecular diagnosis for bovine tuberculosis to improve diagnostic schemes. PloS ONE. (2018) 13:e0207614. 10.1371/journal.pone.0207614 30475835 PMC6261039

[21] Wangoo A Johnson L Gough J Ackbar R Inglut S Hicks D. Advanced granulomatous lesions in Mycobacterium bovis-infected cattle are associated with increased expression of type I procollagen, γδ (WC1+) T cells and CD 68+ cells. J Comp Pathol. (2005) 133:223–34. 10.1016/j.jcpa.2005.05.001 16154140

[22] García-Jiménez WL Salguero FJ Fernández-Llario P Martínez R Risco D Gough J. Immunopathology of granulomas produced by Mycobacterium bovis in naturally infected wild boar. Vet Immunol Immunopathol. (2013) 156:54–63. 10.1016/j.vetimm.2013.09.008 24144683

[23] Johnson LK Liebana E Nunez A Spencer Y Clifton-Hadley R Jahans K. Histological observations of bovine tuberculosis in lung and lymph node tissues from British deer. Vet J. (2008) 175:409–12. 10.1016/j.tvjl.2007.04.021 17584504

[24] Sánchez-Carvajal J Vera-Salmoral E Cuéllar-Gómez R Galán-Relaño A Carrasco L Ruedas-Torres I. Evaluation of droplet digital PCR targeting IS6110 to detect *Mycobacterium tuberculosis complex* DNA in microbiological culture and fresh tissue samples. Proceedings of the the 5th Congress of the European Association of Veterinary Laboratory Diagnosticians (EAVLD). (2021) Nov, virtual meeting.

[25] Good M Bakker D Duignan A Collins DM. The history of *in vivo* tuberculin testing in bovines: Tuberculosis, a “One Health” issue. Front Vet Sci. (2018) 5:59. 10.3389/fvets.2018.00059 29686992 PMC5900347

[26] Thoen CO RG Barletta. Pathogenesis of Mycobacterium bovis. In: Thoen CO Steele JH Glisdorf M.J, editors. Mycobacterium bovis Infection in Animals and Humans. 2a ed. United States: Blackwell Publishing (2006). p. 18–33.

[27] Caswell JL KJ Williams. Respiratory system. In: Maxie M, editor. Pathology of Domestic Animals, Vol 2. 6th ed. Philadelphia: Elsevier Limited (2016). p. 591.

[28] Corner LAL Murphy D Gormley E. Mycobacterium bovis infection in the eurasian badger (Meles meles): the disease, pathogenesis, epidemiology and control. J Comp Pathol. (2011) 144:1–24. 10.1016/j.jcpa.2010.10.003 21131004

[29] Cardoso-Toset F Gómez-Laguna J Amarilla SP Vela AI Carrasco L Fernández-Garayzábal JF. Multi-Etiological nature of tuberculosis-like lesions in condemned pigs at the slaughterhouse. PLoS ONE. (2015) 10:e0139130. 10.1371/journal.pone.0139130 26418681 PMC4587938

[30] Pollock JM Neill SD. Mycobacterium bovis infection and tuberculosis in cattle. Vet J. (2002) 163:115–27. 10.1053/tvjl.2001.0655 12093187

[31] Cassidy J.P. The pathology of bovine tuberculosis: time for an audit. Vet J. (2008) 176:263–4. 10.1016/j.tvjl.2007.09.001 17936047

[32] Gómez-Laguna J Carrasco L Ramis G Quereda JJ Gómez S Pallarés FJ. Use of real-time and classic polymerase chain reaction assays for the diagnosis of porcine tuberculosis in formalin-fixed, paraffin-embedded tissues. J Vet Diagnostic Investig. (2010) 22:123–7. 10.1177/104063871002200126 20093700

[33] Gutiérrez Cancela MM García Marín JF. Comparison of Ziehl-Neelsen staining and immunohistochemistry for the detection of Mycobacterium bovis in bovine and caprine tuberculous lesions. J Comp Pathol. (1993) 109:361–70. 10.1016/S0021-9975(08)80299-X 7508954

[34] Palmer MV Waters WR Thacker TC. Lesion development and immunohistochemical changes in granulomas from cattle experimentally infected with Mycobacterium bovis. Vet Pathol. (2007) 44:863–74. 10.1354/vp.44-6-863 18039899

[35] Menin Á Fleith R Reck C Marlow M Fernandes P Pilati C. Asymptomatic cattle naturally infected with Mycobacterium bovis present exacerbated tissue pathology and bacterial dissemination. PLoS ONE. (2013) 8:18–21. 10.1371/journal.pone.0053884 23326525 PMC3541226

[36] Buddle BM Livingstone PG De Lisle GW. Advances in ante-mortem diagnosis of tuberculosis in cattle. N Z Vet J. (2009) 57:173–80. 10.1080/00480169.2009.36899 19649010

